# A Rare Case of Morquio Syndrome in Palestine: Clinical, Radiological, and Genetic Insights

**DOI:** 10.7759/cureus.82676

**Published:** 2025-04-21

**Authors:** Rou’a E Farah, Rahaf E Farah, Isra W Ayasa, Shadi Abuisneina

**Affiliations:** 1 College of Medicine and Health Sciences, Palestine Polytechnic University, Hebron, PSE; 2 Pediatric Orthopedics, Princess Alia Governmental Hospital, Hebron, PSE

**Keywords:** genetics, lysosomal storage disorder, morquio a syndrome, mps iva, mucopolysaccharidosis type iva, skeletal abnormalities

## Abstract

Morquio A syndrome, also known as mucopolysaccharidosis type IVA (MPS IVA), is a rare lysosomal storage disorder that mostly affects the skeletal system. It spares cognitive function but causes short stature, increasing deformities and anomalies in the joints, as well as respiratory, cardiac, dental, hearing, and vision problems.

This report describes a six-year-old boy with growth retardation and skeletal deformities, and highlights the characteristic clinical features, diagnostic approach, and challenges in the management of MPS IVA, emphasizing the importance of early recognition and comprehensive treatment strategies to optimize patient outcomes.

## Introduction

Mucopolysaccharidosis type IVA (MPS IVA), also known as Morquio A syndrome (OMIM 253000), is a rare, autosomal recessive lysosomal storage disease. It arises from a deficiency of the enzyme N-acetylgalactosamine-6-sulfate sulfatase (*GALNS*, EC 3.1.6.4). This deficiency leads to the accumulation of glycosaminoglycans (GAGs), particularly keratan sulfate (KS) and chondroitin-6-sulfate (C6S), in various tissues, including bone, cartilage, heart valves, and the cornea. The buildup of these substances causes severe systemic skeletal dysplasia, with incomplete ossification resulting in growth impairment [1]. The estimated prevalence of MPS IVA ranges from one in 76,000 to one in 640,000 births [2].

Infants with Morquio A syndrome typically appear normal at birth. However, as storage material accumulates in tissues and organs, causing cellular dysfunction, they progressively develop a variety of serious and debilitating health issues. These include skeletal and joint abnormalities, short stature, respiratory function impairments (both restrictive and obstructive lung disease), cardiac issues (such as valvular regurgitation and stenosis, low stroke volume), as well as hearing and vision problems. Additionally, they may face abdominal manifestations (like hepatomegaly and hernias) and dental abnormalities, while typically maintaining normal intelligence [3,4].

The diagnostic approach for MPS IVA begins with clinical suspicion, often informed by clinical signs and skeletal radiographs. With the implementation of pilot or routine newborn screening (NBS) programs, presymptomatic neonates can also be identified early due to low *GALNS* enzyme activity detected in dried blood spots. In both cases, the diagnosis of MPS IVA is confirmed through an enzyme assay measuring *GALNS* activity in leukocytes or fibroblasts, followed by molecular analysis [5].

Treatment of Morquio A syndrome has traditionally focused on supportive care, aiming to manage symptoms through physical therapy and surgical interventions [6].

Conventional enzyme replacement therapy (ERT) and hematopoietic stem cell transplantation (HSCT) are currently options for patients with MPS IVA. However, there is no definitive treatment available that significantly affects bone and cartilage lesions [2].

## Case presentation

**Figure 1:**
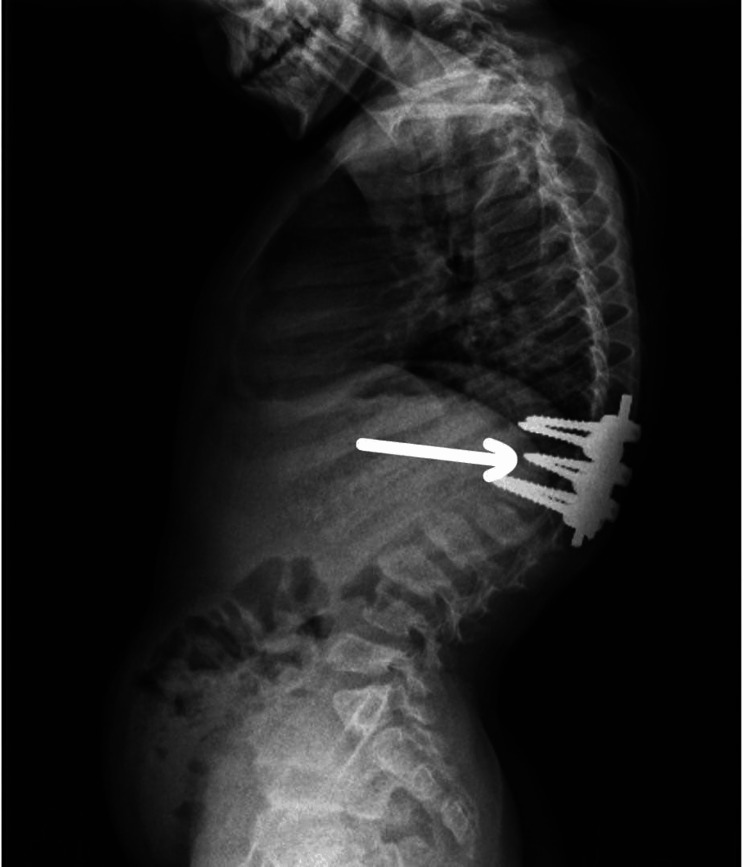
Lateral X-ray view: Postoperative image demonstrating spinal fixation in a patient with Morquio syndrome, showing severe kyphosis and vertebral abnormalities characteristic of the disorder.

**Figure 2:**
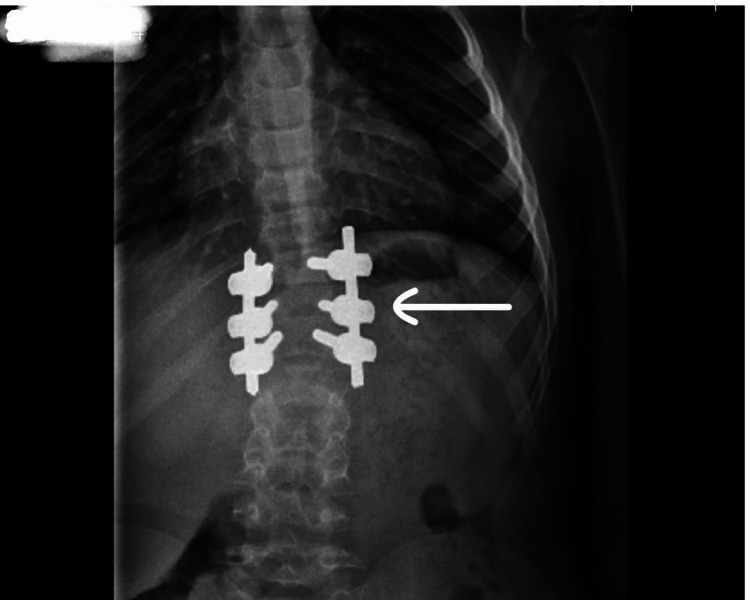
Anteroposterior X-ray view: Postoperative image showing spinal fixation with metal implants in a patient with Morquio syndrome, demonstrating vertebral abnormalities and scoliosis correction.

A six-year-old boy born to consanguineous parents (second-degree relatives) presented with growth retardation and progressive skeletal deformities, which were first noticed at the age of five years. The parents, aged 20 (mother) and 28 (father) at conception, have no family history of a similar condition. This is the first case of Morquio syndrome in the family. This patient was born as a product of an uneventful pregnancy with normal ultrasonography and perinatal complications. The boy had no abnormal features at birth and achieved developmental milestones on time. By five years, he was of short stature, and skeletal deformities were noticed. Clinical examination revealed a pigeon chest, retrognathia, and progressive scoliosis for which surgical fixation was done by six years of age. Imaging demonstrated a classic tailed vertebral column (Figures 1, 2).

There were identified craniofacial anomalies, including hypertelorism and retrognathia, and ocular findings of hypermetropia. A heart murmur was noted on cardiovascular assessment, and the echo showed mitral regurgitation. Genetic analysis using whole-exome sequencing (WES) identified a homozygous pathogenic variant of the *GALNS* gene (p.A492T), previously reported in association with mucopolysaccharidosis type IVA (Morquio syndrome) NM_000512.4: exon13: c.1474G>A: P.A492T. This variant did not show up in general population databases, which makes it less likely to be a benign polymorphism, as was also strongly predicted from computational evidence. This mutation was found in ClinVar (Variation ID: 800974) and assessed as likely pathogenic. Family genetic testing provided additional insights. According to the analysis of family genetic testing, both parents and a younger three-year-old sister were found to be carriers. The sister of the patient, six years of age, was also affected by the disease.

## Discussion

MPS IVA is caused by a likely pathogenic variant in the *GALNS* gene, which encodes N-acetylgalactosamine-6-sulfate sulfatase. This enzyme plays a crucial role in degrading keratan sulfate (KS) and chondroitin-6-sulfate (C6S). This deficiency results in the buildup of these GAGs within lysosomes, leading to various cellular dysfunctions and tissue damage [1]. One of the most obvious phenotypes of this defect is the skeletal system, which is characteristically affected by dysostosis multiplex. This includes short stature due to impaired bone growth, scoliosis and kyphosis due to vertebral abnormalities, and pectus carinatum and retrognathia due to rib and mandibular deformities.

Other systemic manifestations include cardiac involvement, such as valvular regurgitation and stenosis due to GAG deposition in heart valves [3], ophthalmological issues, such as corneal clouding and hypermetropia, respiratory complications, such as restrictive and obstructive lung disease due to skeletal deformities and airway narrowing [5], and dental abnormalities, such as thin enamel and widely spaced teeth.

The diagnosis of MPS IVA is based on a combination of clinical findings, biochemical testing, and genetic analysis. Clinical suspicion arises from features such as skeletal abnormalities, short stature, and multi-organ involvement. Biochemical confirmation involves an enzyme assay to measure *GALNS* activity in leukocytes or fibroblasts, which is typically reduced or absent in affected individuals [4,7]. Molecular analysis further supports the diagnosis by identifying pathogenic variants in the *GALNS* gene, such as the p.A492T variant observed in this case. Additionally, the implementation of newborn screening (NBS) programs has revolutionized early diagnosis by detecting low *GALNS* enzyme activity in dried blood spots, allowing for presymptomatic identification and early intervention [5].

The management of MPS IVA is multidisciplinary and includes both supportive and disease-modifying therapies, including physical therapy to maintain joint mobility and muscle strength; surgical interventions for skeletal deformities (e.g., scoliosis surgery) and cardiac valve repair; respiratory support such as non-invasive ventilation for obstructive sleep apnea; ophthalmological care such as corrective lenses for refractive errors; and disease-modifying therapies such as enzyme replacement therapy (ERT). Elosulfase alfa (Vimizim) is the only FDA-approved ERT for MPS IVA. It aims to reduce GAG accumulation and improve endurance and respiratory function. However, ERT has limited efficacy in addressing bone and cartilage lesions [4]. Hematopoietic stem cell transplantation (HSCT) has been explored as a treatment option, but its benefits are controversial, particularly for skeletal manifestations [3]. Preclinical studies are investigating the use of gene therapy to restore *GALNS* enzyme activity [1]. Substrate reduction therapy (SRT) aims to reduce GAG synthesis, potentially complementing ERT.

Given the autosomal recessive inheritance pattern of MPS IVA, genetic counseling is essential for affected families, including carrier testing for at-risk family members, prenatal diagnosis for future pregnancies using chorionic villus sampling (CVS) or amniocentesis, and preimplantation genetic diagnosis (PGD) for families seeking to avoid passing on the condition [5].

## Conclusions

This case highlights the importance of early diagnosis and a multidisciplinary approach to managing MPS IVA. While current therapies, such as ERT, provide some clinical benefits, they do not fully address the skeletal and cartilage manifestations of the disease. Emerging therapies, including gene therapy and SRT, hold promise for more effective treatment in the future. Genetic counseling and family testing are critical to preventing recurrence and providing informed reproductive options for affected families.
